# Oral condition at admission predicts functional outcomes and hospital-acquired pneumonia development among acute ischemic stroke patients

**DOI:** 10.1007/s00784-024-05833-w

**Published:** 2024-07-19

**Authors:** Futoshi Eto, Tomohisa Nezu, Hiromi Nishi, Shiro Aoki, Saki Tasaka, Susumu Horikoshi, Kanako Yano, Hiroyuki Kawaguchi, Hirofumi Maruyama

**Affiliations:** 1 Department of Clinical Neuroscience and Therapeutics, Graduate School of Biomedical and Health Sciences, Hiroshima University, 1-2-3 Kasumi, Minami ward, Hiroshima, 734-8551 Japan; 2 Department of General Dentistry, Hiroshima University Hospital, Hiroshima, Japan; 3 Division of Dental, Department of Clinical Practice and Support, Hiroshima University Hospital, Hiroshima, Japan

**Keywords:** Acute ischemic stroke, Hospital-acquired pneumonia, Medical-dental integration, Modified oral assessment grade

## Abstract

**Introduction:**

Oral care is crucial for the prevention of cardiovascular events and pneumonia. However, few studies have evaluated the associations between multidimensional assessments of oral status or functional outcomes and hospital-acquired pneumonia (HAP).

**Methods:**

Consecutive patients with acute ischemic stroke (AIS) were retrospectively analyzed. We evaluated the modified oral assessment grade (mOAG) and investigated its association with a modified Rankin scale (mRS) score of 0‒2 (good stroke outcome) and HAP. The mOAG was developed to evaluate 8 categories (lip, tongue, coated tongue, saliva, mucosa, gingiva, preservation, and gargling) on a 4-point scale ranging from 0 to 3. We analyzed the effectiveness of the mOAG score for predicting stroke outcome or HAP using receiver operating characteristic (ROC) curve analysis.

**Results:**

In total, 247 patients with AIS were analyzed. The area under the ROC curve of the mOAG for predicting poor outcomes was 0.821 (cutoff value: 7), and that for HAP incidence was 0.783 (cutoff value: 8). mOAG (a one-point increase) was associated with poor stroke outcome (odds ratio [OR] 1.31, 95% confidence interval [CI] 1.17‒1.48, *P* < 0.001) and HAP (OR 1.21, 95% CI 1.07‒1.38, *P* = 0.003) after adjusting for baseline clinical characteristics, including age and stroke severity.

**Conclusions:**

Lower mOAG scores at admission were independently associated with good outcomes and a decreased incidence of HAP. Comprehensive oral assessments are essential for acute stroke patients in clinical settings.

**Supplementary Information:**

The online version contains supplementary material available at 10.1007/s00784-024-05833-w.

## Introduction

Oral care is important for stroke patients both before and after stroke development. A 91% prevalence of poor oral status has been reported for patients admitted to an acute care hospital [1]. The effectiveness of oral care is well known based on several studies, with reports describing reductions in the occurrence of pneumonia [2] and improvements in the oral environment and intake capacity [3].

Recently, some reports on oral status prior to hospitalization have also been published. There have been many reports on periodontal disease (PD) because it is significantly associated with cerebrovascular disease (CVD) [4]. PD is associated with atrial fibrillation (AF) [5], intracranial artery stenosis [6], obesity [7], risk of CVD regardless of sex [8], and cerebral microbleeds caused by specific bacteria [9]. Although these studies focused on the effect of PD, few studies have elucidated the associations between multidimensional assessments of oral status or functional outcome at 3 months after stroke onset and hospital-acquired pneumonia (HAP).

We reported a reduction in HAP among acute stroke patients achieved through the implementation of a multidisciplinary swallowing team [10]. When acute stroke patients are admitted to our hospital, dentists and dental hygienists perform screenings and provide regular dental care to patients who require continuous care to preserve their oral environment. In our hospital, we implemented the modified oral assessment grade (mOAG) to assess the oral health status of in-hospital patients with various disorders, including stroke, other neurological diseases, cancer, and cardiovascular diseases, in 2017. The mOAG score differs from the revised oral assessment grade (ROAG) [11], a traditional tool for evaluating oral status, in 3 ways. First, a 4-point scale is used for each category. Second, 2 categories related to the tongue are included. Third, “gargling” is an additional category, whereas “voice” and “swallowing” are not included. In this study, we used this quantitative score to assess the oral environment in the early phase of hospitalization and investigated its associations with outcomes 3 months after onset and HAP incidence.

## Methods

### Study design and patients

We conducted this single-center, hospital-based, retrospective study of consecutive patients with acute ischemic stroke (AIS) who were hospitalized at Hiroshima University Hospital.

All AIS patients who were admitted to our institutes within 7 days of symptom onset or who were last known well were prospectively registered in our registry. The data from July 2017 to August 2023 were retrospectively reviewed, and patients who met the following criteria were included: (1) admitted with AIS and (2) had available medical history and laboratory data on admission.

### Clinical data collection

Baseline data on the following variables were collected from the registry: sex, age, body mass index (BMI), smoking status (current or noncurrent smokers), daily alcohol intake (> 40 g/day), antithrombotic therapy before the onset of ischemic stroke, and medical history such as hypertension, dyslipidemia, diabetes mellitus (DM), AF, ischemic heart disease (IHD), chronic heart failure (CHF), chronic kidney disease (CKD), and stroke.

Hypertension was diagnosed if the patient’s blood pressure was ≥ 140/90 mmHg [12] or if the patient had received any antihypertensive medication. Dyslipidemia was diagnosed if the patient had low-density lipoprotein cholesterol ≥ 140 mg/dL, triacylglycerols ≥ 150 mg/dL, or/or high-density lipoprotein cholesterol < 40 mg/dl according to the criteria established by the Japan Atherosclerosis Society [13] or if the patient had a medical history of hypercholesterolemia. DM was diagnosed based on a fasting serum glucose ≥ 126 mg/dL, a serum glucose ≥ 200 mg/dL on 2 random measurements, and a glycated hemoglobin ≥ 6.5% [14] or a medical history of DM. Patients were classified as either current or noncurrent smokers. AF was diagnosed with an electrocardiogram performed previously or on admission. The diagnosis of CHF was made in accordance with the judgment of the attending physician. IHD is a general term for coronary artery disease, myocardial ischemia, and myocardial infarction. CKD was defined as a reduction in the estimated glomerular filtration rate to < 60 ml/min/1.73 m^2^ [15]. Stroke was defined as ischemic stroke, transient ischemic attack, hemorrhagic stroke, or subarachnoid hemorrhage.

Furthermore, we included data such as the premorbid modified Rankin scale (mRS) and National Institutes of Health Stroke Scale (NIHSS) scores at admission, stroke etiology, laboratory findings (hemoglobin, albumin, C-reactive protein (CRP)), and HAP. Laboratory findings were obtained on the day of admission. The mRS and NIHSS scores were defined as described previously [16, 17]. Stroke etiology was classified by stroke neurologists according to the Trial of ORG 10172 in the Acute Stroke Treatment criteria [18]. HAP results from the development of lung infection after at least 48 h of hospitalization [19].

### Assessment of oral environments

**Fig. 1:**
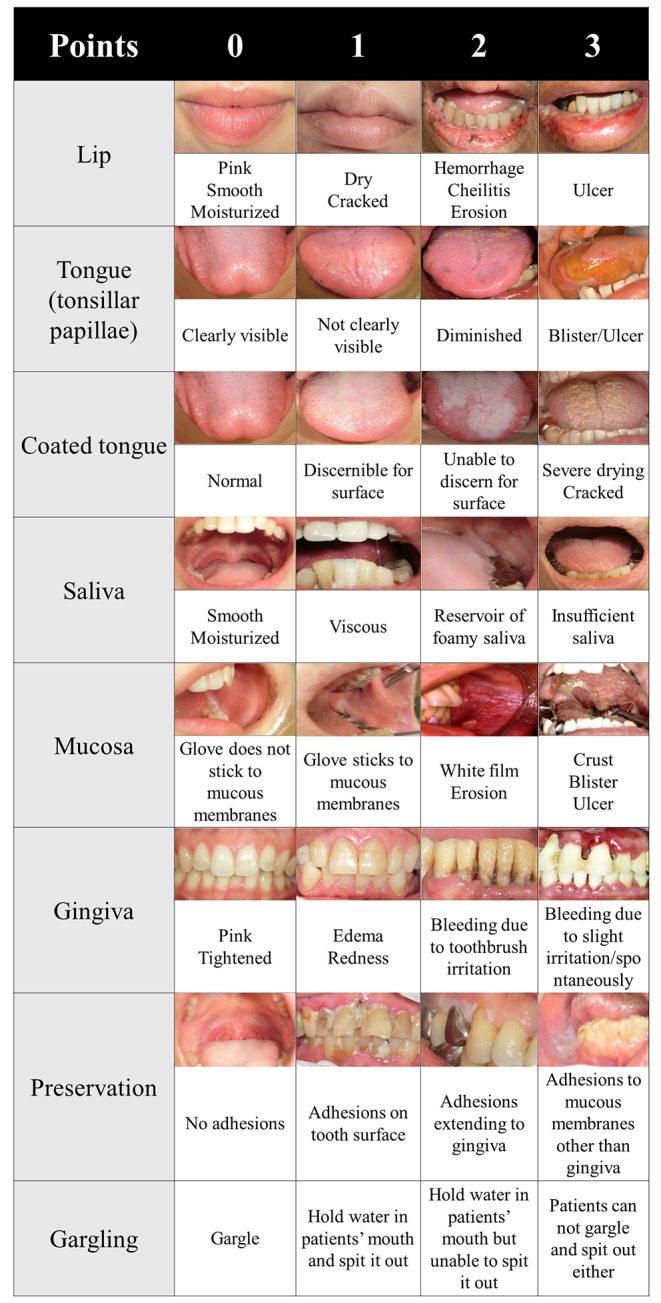
Modified oral assessment grade consists of 8 categories with 4 points. A higher number of categories with scores in the range of 0 to 3 indicates a greater likelihood of a poor oral environment in each category. The categories included lip, tongue, coated tongue, saliva, mucosa, gingiva, preservation, and gargling

In this study, we applied the mOAG score used in our hospital as an index of oral conditions (Fig. 1). The mOAG evaluates 8 categories (lip, tongue, coated tongue, saliva, mucosa, gingiva, preservation, and gargling) on a 4-point scale ranging from 0 to 3. The total score ranges from 0 to 24, and higher scores indicate a poorer oral environment. Dentists and dental hygienists prospectively evaluated these 8 categories within 3 days after hospitalization. The number of teeth lost, excluding wisdom teeth, was also recorded. Standard dental care was provided to hospitalized stroke patients who could not visit the dental clinic and was administered bedside. We provided essential dental care within the ward, including teeth cleaning with toothbrushes, tongue cleaning using tongue brushes, and the use of auxiliary tools such as dental floss to remove plaque, aiming for plaque-free oral hygiene as a critical aspect of care. All dental procedures were standardized in frequency and technique, ensuring consistent patient care. The scheduling of dental interventions was based on the mOAG score, which typically occurs every 2 to 5 days. For patients with lower mOAG scores and those without consciousness impairments, oral hygiene guidance was provided to educate and empower them to maintain their oral health. Once outpatient visits became possible, scaling to remove tartar and teeth polishing with rubber cups were conducted. Oral care was continued until discharge, allowing for continuous monitoring of improvements in oral health throughout the hospitalization.

### Assessment of stroke outcomes

The stroke outcome was evaluated as the 3-month functional status. We assessed a good outcome as an mRS score of 0–2 and a poor outcome as a score of 3–6. In summary, attending physicians evaluated the mRS score at 3 months after stroke onset by examining each patient. When the physicians could not examine a patient, we assessed the mRS score based on a review of the medical records or by contacting the patient or their caregiver/the rehabilitation hospital. Joint assessments were performed for consensus by stroke neurologists (F.E. and T.N.) if needed.

### Statistical analyses

All patient data were categorized into 2 groups and analyzed using the Mann‒Whitney *U* test for continuous data and the two-sided Fisher’s exact test for categorical data. Continuous variables are presented as medians (interquartile ranges). Categorical variables are presented as frequencies and percentages. The patients were divided into tertiles according to the number of subjects in order of mOAG (lowest tertile, middle tertile and highest tertile). The statistical significance of intergroup differences was assessed using the *χ*^*2*^ test and Kruskal‒Wallis test, as appropriate. Multiple linear regression revealed indicators of the severity of mOAG, including age, sex, BMI, smoking status, daily alcohol intake, antithrombotic therapy status, comorbidities (hypertension, dyslipidemia, DM, AF, IHD, CHF, and CKD), history of stroke, premorbid mRS score, NIHSS score at admission, onset to admission time and blood laboratory findings (hemoglobin, albumin, and CRP), according to a backward selection procedure, and *P* > 0.10 for the likelihood ratio test was used as the exclusion criterion. Receiver operating characteristic (ROC) curves were generated to obtain the cutoff values for the mOAG as a factor predicting outcome, and the area under the curve (AUC) for each index to predict a good outcome (mRS score of 0–2 at 3 months) or HAP was calculated. mOAG was evaluated in 2 ways: data were compared with continuous ranges and 2 groups were divided using the cutoff value.

Multivariate logistic analysis was performed to identify the predictors of good stroke outcome or HAP according to the baseline characteristics, including sex, age, BMI, smoking status, daily alcohol intake, antithrombotic therapy, comorbidities (hypertension, dyslipidemia, DM, AF, IHD, CHF, and CKD), history of stroke, NIHSS scores at admission, onset to admission time, blood laboratory findings (hemoglobin, albumin, and CRP), and number of teeth lost, with a backward selection procedure using *P* > 0.10 for the likelihood ratio as the exclusion criterion. Significance was defined as *P* < 0.05 for all tests. Model 1 investigated the situation in which mOAG was analyzed as a continuous variable, whereas Model 2 examined mOAG by dividing patients into 2 groups based on a cutoff value. All analyses were performed using JMP 14.0.0 statistical software (SAS Institute, Inc., Cary, NC, USA).

## Results

### Clinical characteristics associated with mOAG

**Fig. 2:**
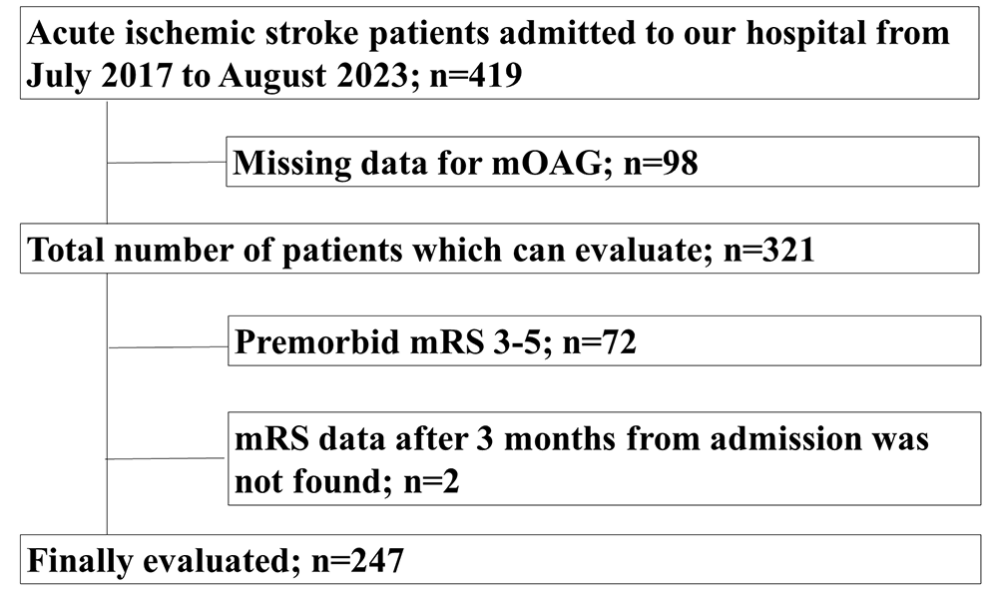
Patient selection process. Initially, 419 patients were included in our registry. Patients were excluded because of a lack of data for mOAG (*n* = 98), lack of stroke outcomes at 3 months (*n* = 2), or premorbid mRS ≥ 3 (*n* = 72). Analysis was performed on the remaining 247 patients Abbreviations: mOAG, modified oral assessment grade; mRS, modified Rankin scale

The flow chart of patient selection is shown in Fig. 2. A total of 419 AIS patients were enrolled in the registry from July 2017 to August 2023. Of these, 98 patients were excluded due to a lack of data on mOAG. Patients who could not undergo mOAG were younger, had lower NIHSS scores at admission, and had fewer teeth lost (Supplemental Table 1). The remaining 321 patients who could be evaluated for mOAG were divided into tertiles based on patient mOAG scores (Table 1). Older age, lower BMI, lower prevalence of daily alcohol intake and greater prevalence of AF and CHF were associated with increased mOAG scores. A history of stroke, a higher premorbid mRS score and a higher NIHSS score at admission were also associated with higher tertiles of mOAG. For the ischemic stroke subtype, the rates of cardioembolic stroke were greater among patients in the higher mOAG tertiles. For laboratory findings, lower albumin levels and higher CRP levels were associated with higher mOAG tertiles. Multiple linear regression revealed that older age (standardized partial regression coefficient [β] 0.164; *P* = 0.001), higher premorbid mRS (β 0.128; *P* = 0.01), CHF (β 0.132; *P* = 0.009), and higher NIHSS score (β 0.413; *P* < 0.01) were independently associated with higher mOAG scores.

**Table 1:** Patient characteristics classified according to modified oral assessment grade (mOAG) tertiles

	mOAG			*P*
	Tertile 1 mOAG 0-5 (*n* = 116)	Tertile 2 mOAG 6-9 (*n* = 105)	Tertile 3 mOAG ≥ 10 (*n* = 100)	
Age	72 [64, 78]	74 [66, 79]	79 [72, 84]	< 0.001
Female	49 (42.2)	40 (38.1)	42 (42.0)	0.788
BMI (kg/m^2^)	22 [20, 25]	22 [20, 25]	21 [18, 23]	0.002
Smoking (*n* = 316)	61 (53.0)	58 (56.3)	61 (62.2)	0.394
Daily alcohol intake (*n* = 316)	27 (23.5)	11 (10.7)	12 (12.2)	0.018
Antithrombotic therapy (*n* = 320)	39 (33.9)	44 (41.9)	39 (39.0)	0.465
Hypertension	86 (74.1)	81 (77.1)	76 (76.0)	0.870
Dyslipidemia	59 (50.9)	53 (50.5)	44 (44.0)	0.539
Diabetes mellitus	29 (25.0)	37 (35.2)	21 (21.0)	0.061
Atrial fibrillation	20 (17.2)	28 (26.7)	40 (40.0)	< 0.001
Ischemic heart disease	16 (13.8)	10 (9.5)	12 (12.0)	0.612
Chronic heart failure	13 (11.2)	16 (15.2)	34 (34.0)	< 0.001
Chronic kidney disease (*n* = 295)	32 (29.6)	30 (32.6)	42 (44.2)	0.077
History of stroke (*n* = 319)	16 (13.9)	32 (30.8)	24 (24.0)	0.009
Premorbid mRS	0 [0, 0]	0 [0, 2]	1.5 [0, 3]	< 0.001
NIHSS on admission (*n* = 315)	2 [1, 5]	5 [3, 13]	15 [6, 26]	< 0.001
Subtype for ischemic stroke				0.002
Atherothrombotic infarction	17 (15.5)	23 (21.9)	15 (15.0)	
Cardioembolic stroke	23 (19.8)	26 (24.8)	43 (43.0)	
Lacunar	19 (16.4)	8 (7.6)	6 (6.0)	
Other etiology	56 (48.3)	48 (45.7)	36 (36.0)	
Intravenous thrombolysis or mechanical thrombectomy	11 (9.5)	14 (13.3)	22 (22.0)	0.034
Onset to admission time (days)	0 [0, 0.8]	0 [0, 0]	0 [0, 0]	0.203
Laboratory findings				
Hb (g/dL) (*n* = 315)	13.1 [11.5, 14.3]	12.5 [10.9, 13.9]	12.3 [10.6, 14.1]	0.088
Alb (g/dL) (*n* = 315)	3.8 [3.3, 4.1]	3.6 [3.1, 3.9]	3.5 [2.9, 3.9]	0.001
CRP (*n* = 311)	0.28 [0.06, 0.81]	0.53 [0.12, 2.45]	0.82 [0.17, 4]	< 0.001
Tooth loss and OAG				
Tooth loss (*n* = 309)	3 [1, 13]	10 [3, 18]	9 [3, 19]	< 0.001
mOAG on admission	3 [2, 4]	6 [5, 7]	11 [10, 14]	< 0.001
Complications				
Hospital-acquired pneumonia	0 (0.0)	5 (4.8)	18 (18.0)	< 0.001

Note: Data are presented as numbers (%) or medians [interquartile ranges]

Abbreviations: Alb, albumin; BMI, body mass index; CRP, C-reactive protein; Hb, hemoglobin; mOAG, modified oral assessment grade; mRS, modified Rankin Scale; NIHSS, National Institutes of Health Stroke Scale

### Clinical course of mOAG

Of the 321 patients with mOAG data, 114 patients (35.5%) were admitted to the intensive care unit. The majority of these patients were transferred to general neurology wards within a few days, where routine medical care, rehabilitation, and oral care were provided. Among the 321 patients with mOAG data, 212 patients (66.0%) were subject to a second mOAG evaluation during hospitalization (median [interquartile ranges]; 8 [7, 14] days). Patients who underwent the second mOAG assessment were older, had a lower BMI, had a lower prevalence of daily alcohol intake and had a greater incidence of AF (Supplemental Table 2). In addition, these patients had higher premorbid mRS scores, higher NIHSS scores on admission and higher initial mOAG scores. The second mOAG score indicated improvements in conditions compared to the initial mOAG score (median, [interquartile ranges]; 7 [4, 10] vs. 8 [5, 11], Wilcoxon matched-pairs signed rank test, *P* < 0.001).

### Associations between mOAG and stroke outcomes

Of the 321 patients with mOAG data, 2 patients were excluded due to a lack of data on their stroke outcomes at 3 months, and 72 patients were excluded due to a preexisting disability with an mRS score ≥ 3 (Fig. 2). Finally, the associations between mOAG and stroke outcome were analyzed in 247 patients. Among them, 137 patients (55.5%) were living independently at 3 months after stroke onset. The baseline characteristics of the patients with mRS scores of 0–2 and those with mRS scores of 3–6 are shown in Table 2. Patients with good outcomes (mRS 0–2) were younger, were mostly males, were not currently smoking, had a lower percentage of AF and CHF, and had a lower NIHSS score. The laboratory findings revealed higher albumin levels and lower CRP levels in these patients compared with n patients → those with poor outcomes. In addition, patients with good outcomes had a lower mOAG score (5 vs. 9, *P* < 0.001) or a lower percentage of mOAG above the cutoff value (25.5% vs. 73.6%, *P* < 0.001), fewer teeth lost (4 vs. 12, *P* < 0.001), and a lower percentage of HAP (0.7% vs. 10.9%, *P* < 0.001).

**Table 2:** Comparisons between patients with modified Rankin Scale scores at 3 months in the cohort

	Modified Rankin Scale		*P*
	0–2 (n = 137)	3–6 (n = 110)	
Age	72 [65, 76]	78 [71, 82]	< 0.001
Female	44 (32.1)	52 (47.3)	0.018
BMI (kg/m^2^)	22.1 [20.1, 24.7]	21.6 [19.6, 24.2]	0.129
Smoking (*n* = 243)	62 (45.6)	72 (67.3)	< 0.001
Daily alcohol intake (*n* = 243)	29 (21.3)	18 (16.8)	0.416
Antithrombotic therapy (*n* = 246)	49 (35.8)	39 (35.8)	1.000
Hypertension	100 (73.0)	92 (83.6)	0.047
Dyslipidemia	71 (51.8)	53 (48.2)	0.61
Diabetes mellitus	36 (26.3)	28 (25.5)	1.000
Atrial fibrillation	22 (16.1)	45 (40.9)	< 0.001
Ischemic heart disease	18 (13.1)	13 (11.8)	0.848
Chronic heart failure	14 (10.2)	24 (21.8)	0.014
Chronic kidney disease (*n* = 229)	38 (29.9)	32 (31.4)	0.886
History of stroke (*n* = 245)	25 (18.3)	20 (18.5)	1.000
NIHSS on admission (*n* = 241)	2 [1, 4]	11 [5, 21]	< 0.001
Subtype for ischemic stroke			< 0.001
Atherothrombotic infarction	25 (18.3)	20 (18.2)	
Cardioembolic stroke	27 (19.7)	44 (40.0)	
Lacunar	23 (16.8)	3 (2.7)	
Other etiology	62 (45.3)	43 (39.1)	
Intravenous thrombolysis or mechanical thrombectomy	12 (8.8)	22 (20.6)	0.015
Onset to admission time (days)	0 [0, 1]	0 [0, 0]	0.614
Laboratory findings			
Hb (g/dL) (*n* = 242)	13.1 [11.5, 14.2]	12.9 [11.1, 14.3]	0.485
Alb (g/dL) (*n* = 242)	3.8 [3.3, 4.1]	3.5 [3.1, 3.9]	0.003
CRP (*n* = 238)	0.28 [0.06, 0.96]	0.71 [0.11, 3.49]	0.001
Tooth loss and OAG			
Tooth loss (*n* = 237)	4 [1, 14]	12 [3, 21]	< 0.001
mOAG on admission (continuous range)	6 [5, 7]	11 [10, 14]	< 0.001
Lip	0 [0, 1]	1 [1, 1]	< 0.001
Tongue (tonsillar papillae)	0 [0, 1]	1 [0, 1]	< 0.001
Coated tongue	0 [0, 1]	1 [1, 1]	< 0.001
Saliva	0 [0, 1]	1 [1, 1]	< 0.001
Mucosa	0 [0, 1]	1 [1, 1]	< 0.001
Gingiva	1 [0, 2]	1 [1, 2]	< 0.001
Preservation	1 [1, 1]	1 [1, 2]	< 0.001
Gargling	0 [0, 0]	3 [0, 3]	< 0.001
mOAG on admission (cutoff value: greater than 7)	35 (25.5)	88 (73.6)	< 0.001
Complications			
Hospital-acquired pneumonia	5 (4.8)	18 (18.0)	< 0.001

Note: Data are presented as numbers (%) or medians [interquartile ranges]

Abbreviations: Alb, albumin; BMI, body mass index; CRP, C-reactive protein; Hb, hemoglobin; mOAG, modified oral assessment grade; mRS, modified Rankin Scale; NIHSS, National Institutes of Health Stroke Scale

**Fig. 3:**
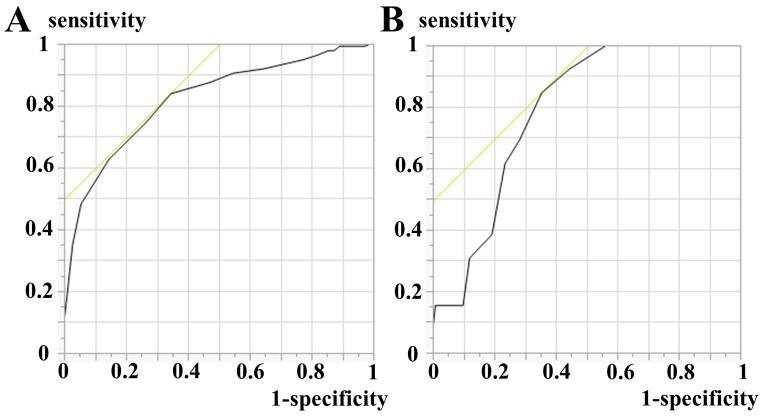
The accuracy of the modified oral assessment grade. (A) ROC curves for mOAG for good outcomes. The vertical axis represents the sensitivity, and the horizontal axis represents the false positive rate (1 - specificity). The optimal cutoff of the mOAG score was 7, which had a sensitivity of 83.9%, a specificity of 65.5%, and an AUC of 0.821. (B) ROC curves for mOAG for HAP. The vertical and horizontal axes are the same as those in Fig. 3A. The optimal cutoff of the mOAG score was 8, which had a sensitivity of 84.6%, a specificity of 64.5%, and an AUC of 0.783 Abbreviations: AUC, area under the receiver operating characteristic curve; HAP, hospital-acquired pneumonia; mOAG, modified oral assessment grade; ROC curves, receiver operating characteristic curves

Multivariable analysis revealed that mOAG (continuous range) was independently associated with poor functional outcome after adjusting for patient background, medical history, laboratory findings, NIHSS score at admission, and tooth loss (odds ratio [OR] 1.31, 95% confidence interval [CI] 1.17–1.48, *P* < 0.001; Table 3, Model 1). The optimal cutoff of the mOAG score for predicting poor outcome was 7, which had a sensitivity of 83.9%, a specificity of 65.5%, and an AUC of 0.821 (Fig. 3A). mOAG (≥ 7) was also associated with poor outcomes (OR 4.26, 95% CI 2.14–8.66, *P* < 0.001; Table 3, Model 2).

**Table 3:** Odds ratio for poor functional outcome at 3 months

	Model 1			Model 2		
	Odds ratio	95% Confidence Interval	*P*	Odds ratio	95% Confidence Interval	*P*
Female	1.96	0.96–4.05	0.065	2.14	1.06–4.39	0.034
Hypertension	3.09	1.26–8.26	0.013	3.14	1.31–8.22	0.01
NIHSS on admission (/1 point)	1.19	1.12–1.28	< 0.001	1.21	1.13–1.30	< 0.001
mOAG (/1 point)	1.31	1.17–1.48	< 0.001			
mOAG (cutoff value: greater than 7)				4.26	2.14–8.66	< 0.001

Model 1 included baseline characteristics, comorbidities, previous stroke episodes, NIHSS scores at admission, other blood laboratory findings (hemoglobin, albumin, and CRP), and number of teeth lost, with a backward selection procedure using *P* > 0.10 as the likelihood ratio as the exclusion criterion. The mOAG score at admission (continuous range) was added to the selection criteria

Model 2: We replaced the mOAG score at admission (continuous range) with the mOAG score at admission (above the cutoff value) as an adjusting factor for the factors selected in Model 1

Abbreviations: mOAG, modified oral assessment grade; NIHSS, National Institutes of Health Stroke Scale

### Associations between mOAG and HAP

Among the 247 patients, 13 (5.3%) had HAP during hospitalization. The baseline characteristics of the patients with HAP are shown in Table 4. A lower percentage of patients with HAP were female, had a medical history of AF, and had higher NIHSS scores. Patients with HAP had lower albumin levels and higher CRP levels compared with those without HAP. In addition, patients who had a higher mOAG score (10 vs. 6, *P* < 0.001) or a greater percentage of mOAG above the cutoff value (84.6% vs. 35.5%, *P* < 0.001) had poorer outcomes → HAP.

**Table 4:** Comparisons between patients with hospital-acquired pneumonia in the cohort

	Hospital-acquired pneumonia		*P*
	yes (*n* = 13)	no (*n* = 234)	
Age	75 [70, 78]	74 [66, 79]	0.895
Female	1 (7.7)	95 (40.6)	0.018
BMI (kg/m^2^)	21.8 [19.2, 23.3]	21.8 [19.9, 24.4]	0.554
Smoking (*n* = 243)	5 (41.7)	129 (55.8)	0.383
Daily alcohol intake (*n* = 243)	2 (16.7)	45 (19.5)	1.000
Antithrombotic therapy (*n* = 246)	8 (61.5)	80 (34.3)	0.071
Hypertension	12 (92.3)	180 (76.9)	0.308
Dyslipidemia	7 (53.9)	117 (50.0)	1.000
Diabetes mellitus	6 (46.2)	58 (24.8)	0.105
Atrial fibrillation	7 (53.9)	60 (25.6)	0.048
Ischemic heart disease	2 (15.4)	29 (12.4)	0.67
Chronic heart failure	3 (23.1)	35 (15.0)	0.429
Chronic kidney disease (*n* = 229)	5 (45.5)	65 (29.8)	0.318
History of stroke (*n* = 245)	3 (25.0)	42 (18.0)	0.466
NIHSS on admission (*n* = 241)	15 [4, 20]	4 [2, 11]	0.019
Subtype for ischemic stroke			0.319
Atherothrombotic infarction	3 (23.1)	42 (18.0)	
Cardioembolic stroke	6 (46.2)	65 (27.8)	
Lacunar	0 (0)	26 (11.1)	
Other etiology	4 (30.8)	101 (43.2)	
Intravenous thrombolysis or mechanical thrombectomy	2 (16.7)	32 (13.9)	0.677
Onset to admission time (days)	0 [0, 0.8]	0 [0, 1]	0.957
Laboratory findings			
Hb (g/dL) (*n* = 242)	13.1 [11.3, 14.0]	13.0 [11.4, 14.3]	0.943
Alb (g/dL) (*n* = 242)	3.3 [2.9, 3.6]	3.6 [3.3, 4.1]	0.025
CRP (*n* = 238)	5.33 [2.58, 9.62]	0.32 [0.08, 1.26]	< 0.001
Tooth loss and OAG			
Tooth loss (*n* = 237)	9 [5, 17]	6 [1, 18]	0.525
mOAG on admission (continuous range)	10 [8, 12]	6 [4, 9]	< 0.001
Lip	1 [1, 1]	1 [0, 1]	0.085
Tongue (tonsillar papillae)	1 [1, 2]	0 [0, 1]	< 0.001
Coated tongue	1 [1, 2]	1 [0, 1]	0.038
Saliva	1 [1, 2]	1 [0, 1]	0.007
Mucosa	1 [1, 2]	0 [0, 1]	0.002
Gingiva	1 [1, 2]	1 [1, 2]	0.204
Preservation	1 [1, 3]	1 [1, 2]	0.064
Gargling	3 [0, 3]	0 [0, 3]	0.015
mOAG on admission (cutoff value: greater than 8)	11 (84.6)	83 (35.5)	0.001
Outcome			
mRS 0–2 at 3 months	1 (7.7)	136 (58.1)	< 0.001

Note: Data are presented as numbers (%) or medians [interquartile ranges]

Abbreviations: Alb, albumin; BMI, body mass index; CRP, C-reactive protein; Hb, hemoglobin; mOAG, modified oral assessment grade; mRS, modified Rankin Scale; NIHSS, National Institutes of Health Stroke Scale

Multivariable analysis revealed that mOAG was independently associated with HAP after adjusting for patient background, medical history, laboratory findings, NIHSS score at admission, and tooth loss (OR 1.21, 95% CI 1.07–1.38, *P =* 0.003; Table 5, Model 1). The optimal cutoff of the mOAG score for predicting HAP incidence was 8, which had a sensitivity of 84.6%, a specificity of 64.5%, and an AUC of 0.783 (Fig. 3B). mOAG (≥ 8) was also associated with HAP incidence (OR 7.89, 95% CI 1.96–52.8; *P =* 0.003; Table 5, Model 2).

**Table 5:** Odds ratio for hospital-acquired pneumonia

	Model 1			Model 2		
	Odds ratio	95% Confidence Interval	*P*	Odds ratio	95% Confidence Interval	*P*
CRP (/1 mg/dl)	1.11	1.02–1.20	0.021	1.10	1.01–1.19	0.032
mOAG (/1 point)	1.21	1.07–1.38	0.003			
mOAG (cutoff value: greater than 8)				7.89	1.96–52.8	0.003

Model 1 included baseline characteristics, comorbidities, previous stroke episodes, NIHSS scores at admission, other blood laboratory findings (hemoglobin, albumin, and CRP), number of teeth lost and mOAG on admission (continuous range), with a backward selection procedure using *P* > 0.10 as the likelihood ratio as the exclusion criterion

Model 2: We replaced the mOAG score at admission (continuous range) with the mOAG score at admission (above the cutoff value) as an adjusting factor for the factors selected in Model 1

Abbreviations: CRP, C-reactive protein; mOAG, modified oral assessment grade

### Associations among mOAG improvements, outcomes, and HAP

Among the 247 patients, 159 patients (64.4%) were subject to a second mOAG evaluation during hospitalization (median [interquartile ranges]; 8 [7, 14] days). Among these 159 patients, 91 experienced improvements in mOAG by at least one point. No association between improvements in the mOAG score and 3-month outcomes were noted (*P* = 0.364). In addition, no association was observed between improvement in mOAG and HAP (*P* = 0.420).

## Discussion

This study demonstrated that mOAG assessments could predict stroke outcomes at 3 months after stroke onset and the development of HAP during hospitalization. Our developed comprehensive oral assessment, mOAG, is potentially effective in the management of acute ischemic stroke patients.

Several medical specialties use visual oral assessment grades and quantify the oral environment to share their findings. The original OAG was developed by nurses to evaluate the oral health status of patients undergoing bone marrow transplantation [20], and the ROAG is an oral health assessment tool for older patients residing in a rehabilitation ward [11]. This tool is useful in identifying the oral conditions that patients complain about [21]. The treatment of stroke patients requires the collaboration of nondental professionals, such as nurses, in addition to dentists and hygienists. The ROAG is beneficial for both dentists and nondentists in assessing oral conditions [22, 23]. This tool also makes it easy to share quality-assured data among multiple professions. Studies employing the ROAG at admission have explored the potential for adverse outcomes associated with decreased swallowing function and malnutrition resulting from sarcopenia [24]. Researchers investigated postacute stroke patients admitted to convalescent rehabilitation wards for oral status, loss of skeletal muscle mass index, and handgrip strength. Poor oral status was associated with reduced muscle mass and strength after adjusting for various factors. Moreover, the correlation between the degree of improvement in the ROAG and outcomes related to dysphagia and physical function during hospitalization was investigated [25]. The researchers evaluated ROAG score changes during hospitalization in convalescent rehabilitation wards and found that a change in the score that was lower than the median value, namely, “good improvement”, was associated with the Functional Independence Measure motor score at discharge. Oral Health Assessment Tool (OHAT) scores are associated with discharge disability, oral intake ability, and longer hospital stays in acute stroke patients older than 65 years [26]. The authors categorized the patients into 2 groups based on their OHAT scores: normal oral health (OHAT score, 0‒2) and poor oral health (OHAT score ≥ 3). The poor oral health group had higher rates of moderate to severe disability at discharge, lower functional oral intake scores, and longer hospital stays.

In the present study, multiple regression analyses revealed that older age, CHF and premorbid mRS were associated with mOAG scores. This result suggested that elderly patients with poor premorbid activities of daily living and heart failure may not be able to perform adequate daily oral care. In addition, the NIHSS score at admission was also associated with mOAG. In these patients, the presence of consciousness disorders or swallowing dysfunction might contribute to poor oral hygiene. We found that mOAG was associated with stroke outcomes. The mOAG was developed for sharing information with nurses, dental hygienists, and dentists in our hospital. It features a 4-level evaluation system, distinguishing itself from the common 3-level evaluations where ‘neither’ is often chosen. Our findings provide further evidence that comprehensive oral assessments are crucial for the acute management of stroke patients. In particular, when a score greater than the cutoff value is observed (specifically, an mOAG score of 7 or higher on admission), it is crucial to communicate the findings among healthcare professionals to implement aggressive oral health care interventions. In the future, it may be valuable to determine which oral assessment methods, such as the mOAG, ROAG, OHAT, or others, are effective in predicting outcomes.

mOAG was independently identified as an associated factor not only with 3-month stroke outcomes but also with HAP. Noguchi et al. reported that OHAT scores were associated with aspiration risk scores in 238 elderly patients with pneumonia. Our results are consistent with insights drawn from the underlying disease and previous reports [27]. The incidence of HAP in this study was 5.3%, which was lower than values of 3.9–12% reported in past reviews and 3.9–28% observed in mixed wards [28]. Notably, a positive trend was noted in our hospital, with the incidence of ischemic stroke and hemorrhagic stroke decreasing from 15.9 to 6.9% in 2016 [10]. This improvement is attributed to our hospital’s implementation of a multidisciplinary swallowing assessment as an integral component of oral care for inpatients. The occurrence rate was further reduced in the 6-year series investigated in this study. One of the reasons for the decreasing trend in HAP at our hospital might be influenced by the impact of introducing mOAG. Although the incidence of HAP decreased, mOAG was still associated with outcomes at 3 months for the following reasons. First, patients with poor oral hygiene are expected to have complications from periodontal disease, which may lead to chronic inflammation and impairment of endothelial cells [29]. Therefore, there is a possibility of poor outcomes due to cardiovascular events, including stroke recurrence. The second reason may be related to malnutrition. Studies of hospitalized patients have reported that oral health, particularly of the lips and mucous membranes, is associated with malnutrition [30]. Several studies have shown that malnutrition is associated with clinical outcomes after stroke [31]. Hence, malnutrition throughout poor oral hygiene might lead to poor stroke outcomes. The oral environment may influence these multiple adverse outcomes, as shown in this study. We believe that sustained intervention by a multidisciplinary swallowing team has played a significant role in influencing the decrease in HAP incidence.

In the present study, a second mOAG assessment was conducted among several severe stroke patients, and the results showed an improving trend. Greater than half of the patients showed improvements in mOAG due to routine oral care. However, no association was observed between mOAG improvement and stroke outcomes or pneumonia. One of the reasons might be that the patients who underwent a second mOAG assessment were predominantly severe cases. Further investigations are needed to determine whether oral care aimed at improving mOAG affects stroke outcomes or HAP.

This study has several limitations. First, the mOAG assessment was not performed in all patients. Patients who were not assessed for mOAG were younger and had a lower NIHSS score and a lower degree of tooth loss (Supplemental Table 1). Therefore, this finding suggests that these individuals exhibit relatively good oral hygiene. However, although the sample size was small, some patients who did not undergo oral assessment still developed HAP, suggesting the possibility of selection bias in the results of this study. Second, we did not prospectively evaluate established oral scores such as ROAG or OHAT. Therefore, a comparison of the merits between mOAG and these scores cannot be made. Although it is important to compare these scoring systems, it is crucial to note that the primary focus of this study was not to establish the superiority of the mOAG compared with established oral scores. Rather, this study emphasizes the significance of a comprehensive oral assessment. We believe that implementing proper oral evaluations and oral care initiatives is important for stroke patients. Third, we could not collect the time (in minutes or hours) from stroke onset to admission. Although onset to admission time (days) was not associated with mOAG, stroke outcomes, or HAP, further detailed analysis limited to patients within 24 h of onset may be necessary in the future. In addition, we did not evaluate socioeconomic factors or daily oral care habits before stroke onset. These factors might influence the mOAG at admission.

## Conclusion

The mOAG score, which reflects the patient’s oral health status at the onset of AIS, is associated with 3-month poststroke outcomes. The assessment of mOAG at admission may also serve as a predictor for developing HAP, offering valuable insights for preventive interventions.

### Electronic supplementary material

Below is the link to the electronic supplementary material.

Supplementary Material 1

## Data Availability

No datasets were generated or analysed during the current study.
