# Supplementary material for: Oral condition at admission predicts functional outcomes and hospital-acquired pneumonia development among acute ischemic stroke patients
Source: Clin Oral Investig. 2024 Jul 19;28(8):434. doi: 10.1007/s00784-024-05833-w (PMC11271429; doi:10.1007/s00784-024-05833-w)
Supplement: Supplementary file 1 — Supplementary Material 1 [file 784_2024_5833_MOESM1_ESM.docx]

**Supplemental Table 1. Comparisons between patients with modified oral assessment grade (mOAG) scores in the cohort.**

|  | **Total (n=419)** | **ⅿOAG** | | ***P*** |
| --- | --- | --- | --- | --- |
|  |  | **Data available (n=321)** | **No data**  **(n=98)** |  |
| Age | 74 [66, 80] | 75 [68, 80] | 72 [32, 78] | 0.044 |
| Female | 170 (40.6) | 131 (40.8) | 39 (39.8) | 0.907 |
| BMI (kg/m^2^) | 21.9 [19.5, 24.7] | 21.8 [19.5, 24.4] | 22.6 [19.5, 25.4] | 0.165 |
| Smoking (n=412) | 226 (54.9) | 180 (57.0) | 46 (47.9) | 0.129 |
| Daily alcohol intake (n=412) | 70 (17.0) | 50 (15.8) | 20 (20.8) | 0.278 |
| Antithrombotic therapy (n=418) | 167 (40.0) | 122 (38.1) | 45 (45.9) | 0.195 |
| Hypertension | 307 (73.3) | 243 (75.7) | 64 (65.3) | 0.05 |
| Dyslipidemia | 206 (49.2) | 156 (48.6) | 50 (51.0) | 0.73 |
| Diabetes mellitus | 118 (28.2) | 87 (27.1) | 31 (31.6) | 0.441 |
| Atrial fibrillation | 113 (27.0) | 88 (27.4) | 25 (25.5) | 0.795 |
| Ischemic heart disease | 52 (12.4) | 38 (11.8) | 14 (14.3) | 0.49 |
| Chronic heart failure | 77 (18.4) | 63 (19.6) | 14 (14.3) | 0.297 |
| Chronic kidney disease (n=392) | 132 (33.7) | 104 (35.3) | 28 (28.9) | 0.267 |
| History of stroke (n=417) | 99 (23.7) | 72 (22.6) | 27 (27.6) | 0.342 |
| Premorbid mRS (n=418) | 0 [0, 2] | 0 [0, 2] | 0 [0, 2] | 0.987 |
| NIHSS on admission | 4 [1, 13] | 5 [2, 14] | 2 [1, 6] | <0.001 |
| Subtype for ischemic stroke |  |  |  | 0.694 |
| Atherothrombotic infarction | 69 (16.5) | 56 (17.5) | 13 (13.3) |  |
| Cardioembolic stroke | 120 (28.6) | 92 (28.7) | 28 (28.6) |  |
| Lacunar | 46 (11.0) | 33 (10.3) | 13 (13.3) |  |
| Other etiology | 184 (43.9) | 140 (43.6) | 44 (44.9) |  |
| Intravenous thrombolysis or mechanical thrombectomy | 55 (13.1) | 47 (14.6) | 8 (8.2) | 0.123 |
| Onset to admission time (days) | 0 [0, 0] | 0 [0, 0] | 0 [0, 0] | 0.247 |
| **Laboratory findings** |  |  |  |  |
| Hb (g/dL) (n=408) | 12.8 [11.1, 14.2] | 12.7 [11.1, 14.1] | 13.1 [11.1, 14.9] | 0.383 |
| Alb (g/dL) (n=405) | 3.6 [3.2, 4.1] | 3.6 [3.2, 4.0] | 3.8 [3.4, 4.1] | 0.028 |
| CRP (n=397) | 0.46 [0.1, 1.81] | 0.52 [0.1, 1.84] | 0.3 [0.1, 1.72] | 0.292 |
| **Tooth loss** |  |  |  |  |
| Tooth loss (n=339) | 6 [1, 17] | 7 [2, 17] | 2 [0, 9] | <0.001 |
| **Complications** |  |  |  |  |
| Hospital-acquired pneumonia | 26 (6.2) | 23 (7.2) | 3 (3.1) | 0.159 |

Note: Data are presented as numbers (%) or medians [interquartile ranges].

Abbreviations: Alb, albumin; BMI, body mass index; CRP, C-reactive protein; Hb, hemoglobin; mOAG, modified oral assessment grade; mRS, modified Rankin Scale; NIHSS, National Institutes of Health Stroke Scale.

**Supplemental** **Table 2. Comparisons between patients with and without modified oral assessment grade (mOAG) follow-up.**

|  | **ⅿOAG** | | ***P*** |
| --- | --- | --- | --- |
|  | **Follow-up**  **(n=212)** | **No follow-up**  **(n=109)** |  |
| Age | 75 [68, 81] | 73 [65, 78] | 0.017 |
| Female | 93 (43.9) | 38 (34.9) | 0.150 |
| BMI (kg/m^2^) | 21 [19, 23] | 23 [20, 26] | <0.001 |
| Smoking (n=316) | 125 (59.5) | 55 (51.9) | 0.229 |
| Daily alcohol intake (n=316) | 20 (9.5) | 30 (28.3) | <0.001 |
| Antithrombotic therapy | 85 (40.3) | 37 (33.9) | 0.278 |
| Hypertension | 161 (75.9) | 82 (75.2) | 0.891 |
| Dyslipidemia | 103 (48.6) | 53 (48.6) | 1.000 |
| Diabetes mellitus | 56 (26.4) | 31 (28.4) | 0.693 |
| Atrial fibrillation | 70 (33.0) | 18 (16.5) | 0.002 |
| Ischemic heart disease | 10 (9.5) | 12 (12.0) | 0.612 |
| Chronic heart failure | 46 (21.7) | 17 (15.6) | 0.235 |
| Chronic kidney disease (n=295) | 64 (33.7) | 40 (38.1) | 0.449 |
| History of stroke (n=319) | 51(24.3) | 21 (19.3) | 0.327 |
| Premorbid mRS | 1.5 [0, 3] | 0 [0, 2] | <0.001 |
| NIHSS on admission | 7 [3, 18] | 3 [1, 5] | <0.001 |
| Subtype for ischemic stroke |  |  | <0.001 |
| Atherothrombotic infarction | 38 (17.9) | 18 (16.5) |  |
| Cardioembolic stroke | 74 (34.9) | 18 (16.5) |  |
| Lacunar | 13 (6.1) | 20 (18.4) |  |
| Other etiology | 87 (41.0) | 53 (48.6) |  |
| Intravenous thrombolysis or mechanical thrombectomy | 36 (17.0) | 11 (10.1) | 0.133 |
| Onset to admission time (days) | 0 [0, 0] | 0 [0, 0.25] | 0.345 |
| **Laboratory findings** |  |  |  |
| Hb (g/dL) (n=315) | 12.4 [10.9, 14] | 13.3 [11.4, 14.7] | 0.039 |
| Alb (g/dL) (n=315) | 3.5 [3.1, 3.9] | 3.7 [3.3, 4.1] | 0.023 |
| CRP (n=311) | 0.7 [0.14, 2.61] | 0.28 [0.07, 0.98] | 0.001 |
| **Tooth loss and OAG** |  |  |  |
| Tooth loss (n=309) | 8 [2, 18] | 5 [1, 17] | 0.198 |
| mOAG on admission | 8 [5, 11] | 5 [3, 7] | <0.001 |
| **Complications** |  |  |  |
| Hospital-acquired pneumonia | 22 (10.4) | 1 (0.9) | 0.001 |

Note: Data are presented as numbers (%) or medians [interquartile ranges].

Abbreviations: Alb, albumin; BMI, body mass index; CRP, C-reactive protein; Hb, hemoglobin; mOAG, modified oral assessment grade; mRS, modified Rankin Scale; NIHSS, National Institutes of Health Stroke Scale.
